# Keratin 19 maintains E-cadherin localization at the cell surface and stabilizes cell-cell adhesion of MCF7 cells

**DOI:** 10.1080/19336918.2020.1868694

**Published:** 2021-01-04

**Authors:** Sarah Alsharif, Pooja Sharma, Karina Bursch, Rachel Milliken, Van Lam, Arwa Fallatah, Thuc Phan, Meagan Collins, Priya Dohlman, Sarah Tiufekchiev, Georges Nehmetallah, Christopher B. Raub, Byung Min Chung

**Affiliations:** a Department of Biology, The Catholic University of America, Washington, District of Columbia, USA; b Department of Biomedical Engineering, The Catholic University of America, Washington, District of Columbia, USA; c Department of Electrical Engineering, The Catholic University of America, Washington, District of Columbia, USA

**Keywords:** Intermediate filaments, keratin, adherens junction, cell-cell adhesion, breast cancer, metastasis, cell migration, cell morphology, keratin 19

## Abstract

A cytoskeletal protein keratin 19 (K19) is highly expressed in breast cancer but its effects on breast cancer cell mechanics are unclear. In MCF7 cells where K19 expression is ablated,*we found that K19 is required to maintain rounded epithelial-like shape and tight cell-cell adhesion. A loss of K19 also lowered cell surface E-cadherin levels. Inhibiting internalization restored cell-cell adhesion of *KRT19** knockout cells, suggesting that E-cadherin internalization contributed to defective adhesion. Ultimately, while K19 inhibited cell migration and invasion, it was required for cells to form colonies in suspension. Our results suggest that K19 stabilizes E-cadherin complexes at the cell membrane to maintain cell-cell adhesion which inhibits cell invasiveness but provides growth and survival advantages for circulating tumor cells.

## Introduction

The majority of cancer deaths are due to metastasis [1–3]. Tumor metastasis is a multistep process that involves spreading of cancer cells from a primary site to colonization at a distal site [2,4]. During its initiation, metastasis has been linked closely to epithelial-to-mesenchymal transition (EMT) whereby cells lose morphological traits of epithelial cells, including tight cell-cell adhesion and apical‐basal polarity, and gain mesenchymal traits such as increased ability to undergo migration and invasion [2,5,6]. At the molecular level, epithelial markers such as E-cadherin and keratins become downregulated while mesenchymal markers such as vimentin become upregulated during EMT.

Keratins belong to an intermediate filament family of cytoskeletal proteins, and keratin filaments maintain epithelial cell polarity and mechanical integrity through intercellular and cell-extracellular matrix junctions called desmosomes and hemidesmosomes, respectively [7,8]. Decreased expression of keratins during EMT is considered to contribute to an initiation of metastasis by loosening cell-cell attachment through disassembly of desmosomes [9–11]. Therefore, it has been suggested that maintenance of intercellular adhesion by keratins serves as a barrier against EMT and cell migration [11], a concept supported by several *in vitro* studies involving keratins expressed in simple epithelium and keratinocytes [5,12–14]. However, upregulation of select keratins has been shown to enhance cell migration and invasion in certain cancer settings [12,15,16], likely due to the fact that some cancer cells invade extracellular matrix collectively [17,18].

Following initiation, metastatic cells intravasate into the bloodstream and must survive in suspension as circulating tumor cells (CTCs) en route to a distal site [2,4,19]. High metastatic potential of CTCs has been associated with stem-like properties [20] and also with clusters of cells with higher levels of cell-cell adhesion molecules plakoglobin or E-cadherin [21,22]. However, the role of keratins on stem-like traits and clustering of cancer cells upon detachment from the extracellular matrix remains unclear.

In the context of breast cancer, previous studies have shown that depletion of K19 increases cell migration and invasion *in vitro*, potentially through upregulation of Akt and Notch signaling pathways [23–25]. However, mammary stem/progenitor cells transformed with sets of oncogenes including mutant Ras and p53 were more metastatic when K19 was present [26]. In addition, the role of K19 in cell-cell mechanics contributing to metastasis-related cancer cell behaviors has not been defined.

To study the role of K19 on processes fundamental to metastasis, we examined the luminal-subtype MCF7 breast cancer cell line which expresses high levels of K19 [27,28]. MCF7 cells maintain polarized epithelial phenotype with intact cell-cell adhesions made of desmosomes and adherens junctions [29–31]. Using MCF7 cells with complete ablation of K19 [32] and *KRT19* knockout (KO) cells with re-expression of K19, we observed that K19 is required for the epithelial-like cell shape and proper cell-cell adhesion. These events were accompanied by lower levels of plakoglobin but accumulation of E-cadherin in endocytic compartments in the absence of K19. Importantly, while we confirmed the inhibitory role of K19 on cell migration and invasion, K19 was found to be required for cells to grow in low attachment conditions.

## Results

### KRT19 KO cells display an elongated phenotype

**Figure 1.**
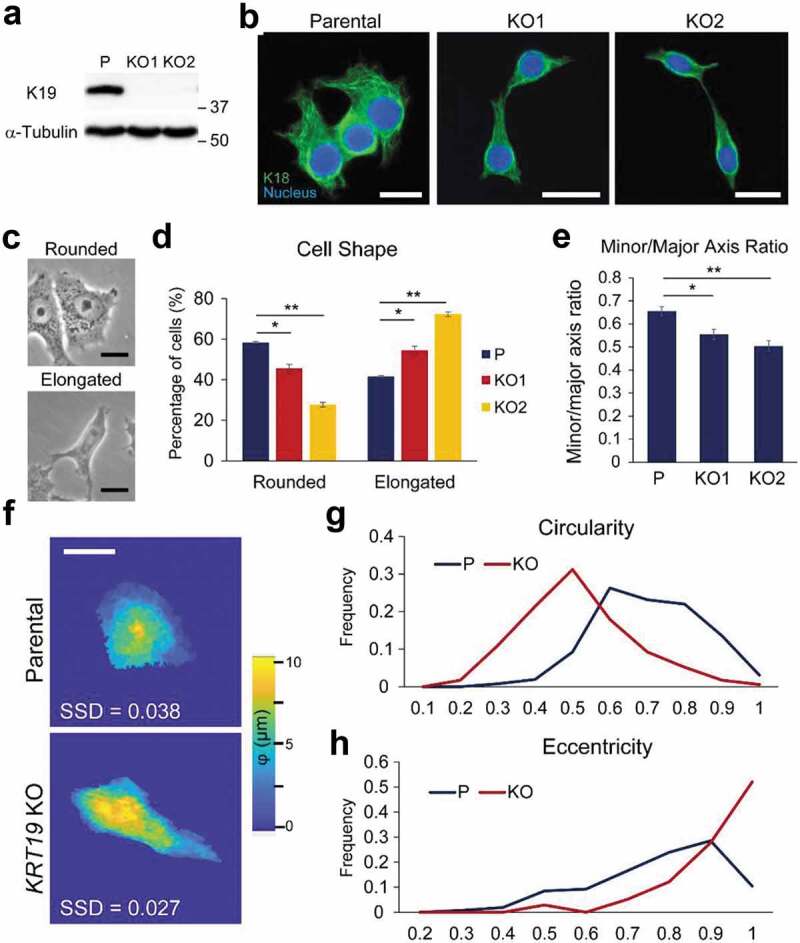
**Keratin 19 knockout cells display an elongated phenotype**. (a) Whole cell lysates of parental (p) control and two different clones (KO1 and KO2) of *KRT19* KO cell lines were harvested, and immunoblotting was performed with antibodies against the indicated proteins. Molecular weights in kDa. (b) Immunostaining of K18 (green) in P and *KRT19* KO cells. Nuclei are shown in blue. Bar, 20 µm. (c) Phase-contrast images of representative rounded and elongated shapes. Bar, 20 µm. (d) Percentages of P and *KRT19* KO cells with rounded or elongated cell shape. Data from three experimental repeats are shown as mean ± SEM. Student’s t-test: *p < 0.05; **p < 0.001. Chi-square test: p < 0.001. (e) Minor/Major axis ratios of P and *KRT19* KO cells. Data from four experimental replicates are shown as mean ± SEM. Student’s t-test: *p < 0.05; **p < 0.001. (f) Phase pseudoheight maps of P and *KRT19* KO (KO2) cells collected by digital holographic microscopy (DHM). Sum of squared deviation (SSD) shows the distance of individual cells from the population mean. Representative P and *KRT19* KO cells with the smallest SSD of 17 phase parameters collected shown. A color bar indicates cell phase height. Bar, 10 µm. Histograms of (g) circularity and (h) eccentricity of P and *KRT19* KO cells from DHM analyses

Under the microscope, MCF7 *KRT19* KO cells showed a considerable difference in morphology from their parental counterpart. While parental (P) MCF7 cells were mostly epithelial-like and rounded in shape, *KRT19* KO cells exhibited more mesenchymal-like morphology with elongated and spindled shapes (Figure 1a–Figure 1b). Of note, two *KRT19* KO clones were used to confirm phenotypes associated with a loss of K19. To quantify the difference in shapes between parental and *KRT19* KO cells, cells were sorted into two categories, rounded and elongated, based on their shapes (Figure 1c). An elongated spindled cell shape with protrusions at cell edges was categorized as elongated while a rounded morphology characterized by a circular cell shape was categorized as rounded. Scoring of cell shapes confirmed that *KRT19* KO cells were more elongated than parental cells as the majority of *KRT19* KO cells (54.4–72.3%) were elongated while the majority of parental cells (58.4%) were rounded in shape (Figure 1d). *KRT19* KO cells also exhibited decreased minor/major axis ratio (0.39–0.44) compared to parental cells (0.50), further verifying more elongated shape (Figure 1e). Circularities of *KRT19* KO cells were also less than that of parental cells (Figure S1A) and when a cutoff value of 0.48 was used for circularity (Figure S1B), the result mirrored what was observed in Figure 1d. Of note, while two *KRT19* KO clones exhibited subtle differences from each other, both were more elongated than parental cells. Finally, digital holographic microscopy (DHM) was used to quantitate morphologies of parental and *KRT19* KO cells (Figure 1f). DHM measured cells based on index of refraction and physical thickness [33,34] and produced 17 parameters per single cell based on individual cell pseudoheight (units in nm) derived from phase maps (Table S1). Optical measurements included pixel mean, standard deviation, and texture parameters, while geometric parameters included roundedness of cells, eccentricity and circularity. A higher eccentricity and a lower circularity frequency confirmed the elongated phenotype of *KRT19* KO cells (Figure 1g–Figure 1h).

### Weakened cell-cell adhesions in KRT19 KO cells

**Figure 2.**
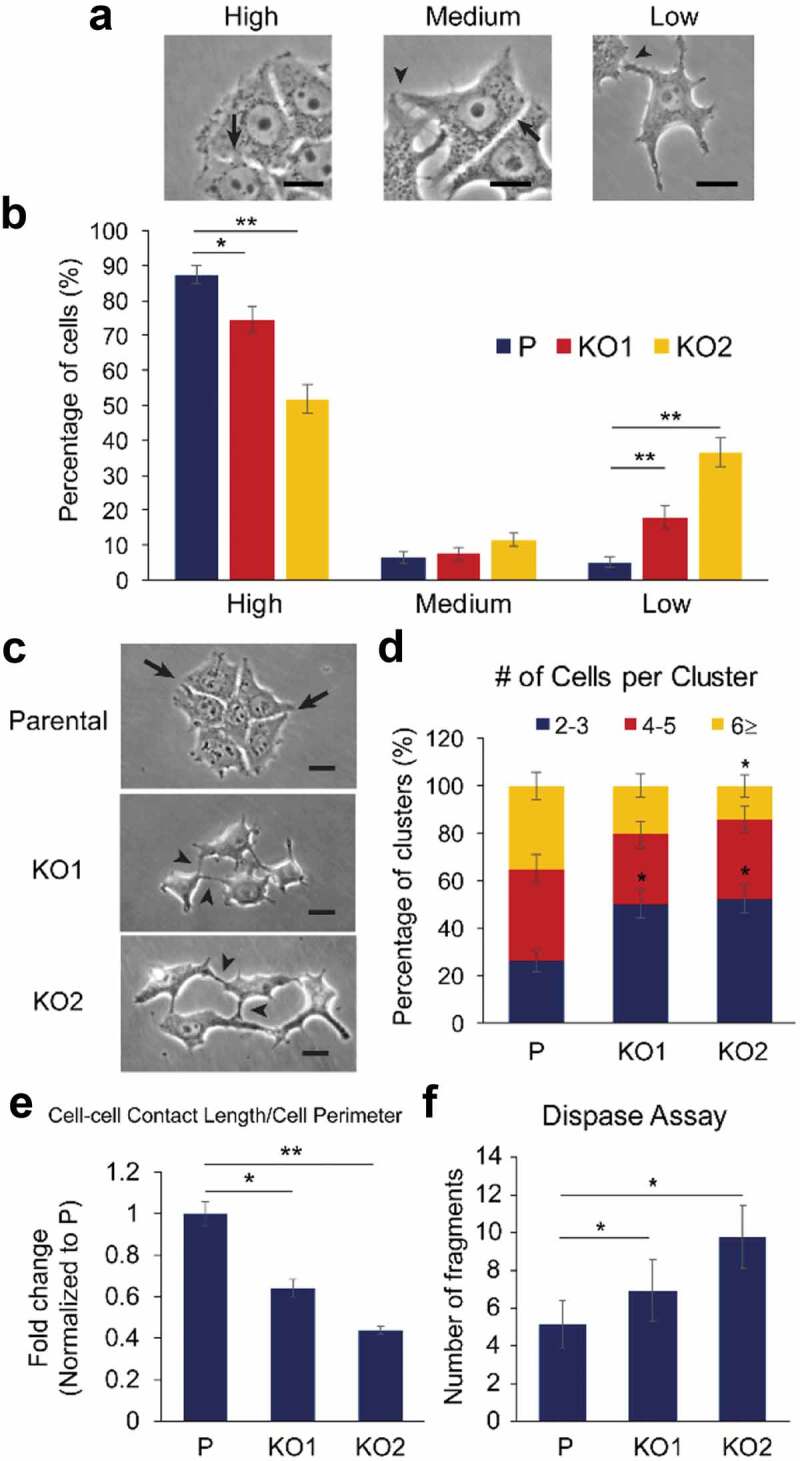
**Weakened cell-cell adhesion in *KRT19* KO cells**. (a) Phase-contrast images of representative cells engaged in high, medium and low adhesions. Arrows indicate high cell-cell adhesions and arrowheads indicate low cell-cell adhesions. Bar, 20 µm. (b) Percentage of parental (p) control and *KRT19* KO cells in high, medium or low adhesions. Data from five experimental repeats are shown as mean ± SEM. Student’s t-test: *p < 0.05; **p < 1 × 0.001. Chi-square test: p < 0.0001. (c) Phase-contrast images of cell clusters formed by P and *KRT19* KO cells. Arrows indicate high cell-cell adhesions and arrowheads indicate low cell-cell adhesions. Bar, 20 µm. (d) Number of cells per cluster. Clusters classified into three different ranges: 2–3 cells/cluster; 4–5 cells/cluster; and >6 cells/cluster. Data from three experimental repeats are shown as mean ± SEM. Any statistically significant difference between P and *KRT19* KO cells is denoted by * (p < 0.05, Student’s t-test) above the cluster of *KRT19* KO cells. Chi-square test: p < 0.0001. (e) Ratio of cell-cell contact length to cell perimeter per cell. Data from four experimental repeats normalized to that of the parental control are shown as mean ± SEM. Student’s t-test: *p < 0.05; **p < 1 × 0.001. (f) Monolayer fragment numbers of P and *KRT19* KO cells from the dispase assay. Data from three experimental repeats, each with three replicates, are shown as mean ± SEM. Student’s t-test: *p < 0.05

In addition to elongated shape, *KRT19* KO cells were forming loose contacts between neighboring cells, whereas parental MCF7 cells were in close contacts with their neighbors (Figure 1b). To quantitate the difference, cell-cell adhesions made by subconfluent parental and *KRT19* KO cells were examined. Cell-cell adhesions were categorized into three different degrees: high indicates a cell attached to its neighboring cells by making contiguous contacts all along adjoining sides; medium indicates a cell attached to its neighbor with contiguous and pointed cell-cell adhesions; and low indicates a cell attached to its neighbor only by pointed cell-cell adhesion (Figure 2a). In the absence of K19, lower percentages of cells exhibited high adhesion (51.9–74.6% vs 88.1% for parental cells), but more cells showed low adhesion (17.9–36.6% vs 5.2% for parental cells), confirming the observation that a loss of K19 resulted in decreased cell-cell adhesion (Figure 2b). Since parental MCF7 cells grow in tight clusters even at low confluency, cell-cell adhesion was also assessed by counting number of cells in each cluster after passaging (Figure 2c). The number of cells making contacts as a group was fewer in *KRT19* KO cells compared to parental cells as 50.0–52.2% of *KRT19* KO cells formed cell clusters with only 2–3 cells while 26.4% of parental cells did so (Figure 2d). In addition, measuring cell-cell contact length and perimeter for each cell showed that *KRT19* KO cells exhibited decreased cell-cell contact length/perimeter ratios compared to parental cells (Figure 2e & S2). Finally, disrupting cell-cell adhesion with dispase treatment and mechanical force induction showed that K19 was required for proper cell-cell adhesion as indicated by a higher number of fragmented monolayers in *KRT19* KO cells (6.9–9.8 vs 5.2 fragments in parental cells, Figure 2f & S3). Collectively, these data support the notion that K19 is required for proper adhesion between cells.

### Decreased cell surface localization of E-cadherin in KRT19 KO cells and defective cell-cell adhesion

**Figure 3.**
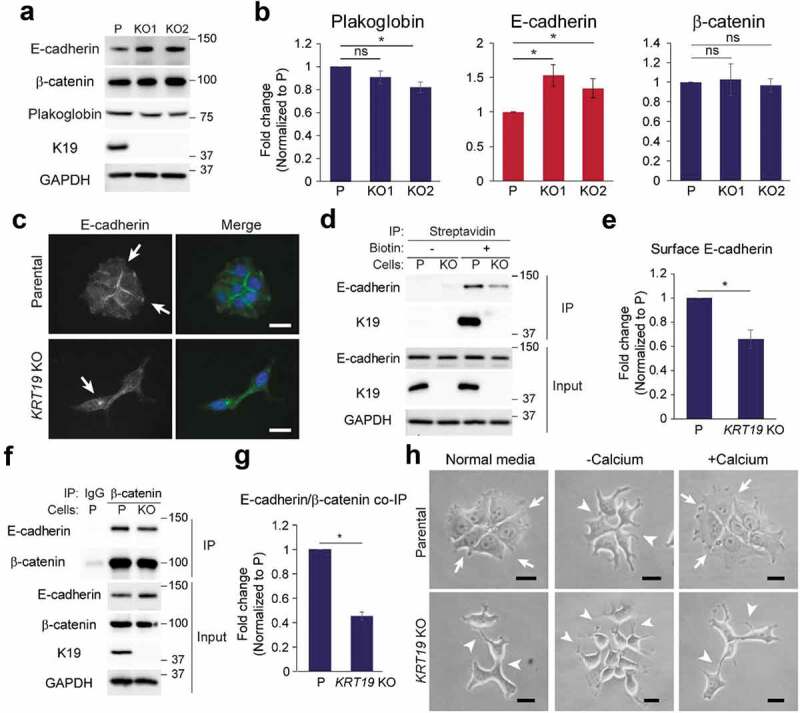
**Defective regulation of E-cadherin in *KRT19 K*O cells**. (a) Whole cell lysates of parental (p) control and *KRT19* KO cells were prepared and immunoblotting was performed with indicated antibodies. Molecular weights in kDa. (b) Signal intensities of plakoglobin, E-cadherin and β-catenin from (**A**) were quantified and normalized to those of the GAPDH loading control. Data from at least three experimental repeats normalized to that of the parental control are shown as mean ± SEM. Student’s t-test: ns: not significant; *p < 0.05. (c) Immunostaining of E-cadherin (green) in P and *KRT19* KO (KO2) cells. Nuclei are shown in blue. Arrows indicate E-cadherin localization. Bar, 20 µm. (d) Streptavidin beads were used to pulldown P and *KRT19* KO (KO2) cells either treated (+) or untreated (-) with biotin for cell surface labeling of proteins. Immunoprecipitates and inputs were subjected to SDS-PAGE and immunoblotting was performed with antibodies against the indicated proteins. Molecular weights in kDa. (e) Signal intensities of E-cadherin in immunoprecipitates of biotin-treated samples from (**D**) were quantified and normalized to those of E-cadherin in input. Data from at least three experimental repeats normalized to that of the parental control are shown as mean ± SEM. Student’s t-test: *p < 0.05. (f) Co-IP of β-catenin with E-cadherin. IP with anti-β-catenin antibody or IgG control was perform in P and *KRT19* KO (KO2) cells. Immunoprecipitates and inputs were subjected to SDS-PAGE and immunoblotting was performed with antibodies against the indicated proteins. Molecular weights in kDa. (g) Signal intensities of E-cadherin in IP from (**F**) were quantified and normalized to those of β-catenin in IP. Data from at least three experimental repeats normalized to that of the parental control are shown as mean ± SEM. Student’s t-test: *p < 0.05. (h) Phase-contrast images of P and *KRT19* KO (KO2) cells. Cells were either grown in normal growth condition (Normal media), placed in calcium-free media for 6 h, then left unstimulated (-Calcium) or stimulated (+Calcium) with CaCl_2_ for 4 h. Arrows indicate high cell-cell adhesions and arrowheads indicate low cell-cell adhesions. Bar, 20 µm

Consistent with decreased cell-cell adhesion, *KRT19* KO cells expressed reduced levels of plakoglobin, a desmosomal and adherens junction component based on RNA-sequencing analyses (Figure 3a–Figure 3b). Surprisingly however, a functional enrichment analysis using Database for Annotation, Visualization and Integrated Discovery (DAVID) showed that many genes upregulated in *KRT19* KO cells were associated with functions in cell membranes, cell adhesions, and cell junctions (Figure S4). Indeed, although levels of β-catenin, a K19-interacting component of adherens junction (Figure S5) remained the same, levels of E-cadherin, a Ca^2+^-dependent adhesive molecule and a key regulator of cell morphology, were found to be increased by 1.53 – and 1.34-folds in *KRT19* KO cells compared to parental cells (Figure 3a–Figure 3b). Despite the increased total levels, the cell surface localization of E-cadherins was decreased in *KRT19* KO cells as assessed by immunostaining of cells (Figure 3c) and surface biotinylation (Figure 3d–Figure 3e). Moreover, quantifying cell-cell adhesion based on E-cadherin staining confirmed that a loss of K19 induced decreased cell-cell adhesion (Figure S6), matching the observation from Figure 2b. Furthermore, interaction between E-cadherin and β-catenin became decreased in *KRT19* KO cells, suggesting a requirement for K19 in proper maintenance of adherens junction (Figure 3f–Figure 3g). Indeed, while stimulating Ca^2+^-deprived parental cells with Ca^2+^ allowed cells to re-adhere to each other, Ca^2+^ stimulation had little effect on adherence of *KRT19* KO cells (Figure 3h), indicating that K19 is required for the formation and function of calcium-dependent junctional complexes. Defective cell-cell adhesion in *KRT19* KO cells was also observed upon serum stimulation (Figure S7).

### Internalization of E-cadherin into endocytic compartments in KRT19 KO cells

**Figure 4.**
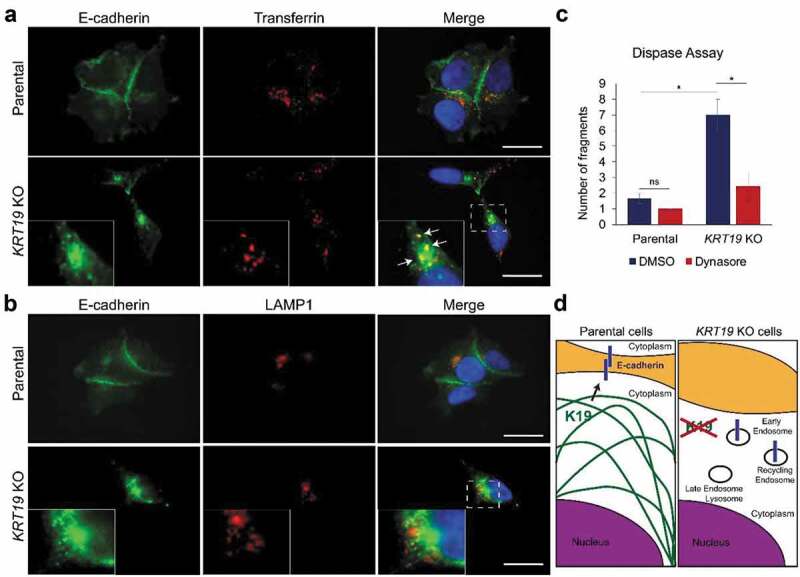
**Inhibiting internalization rescues cell-cell adhesion of *KRT19* KO cells**. Parental and *KRT19* KO (KO2) cells were either (a) incubated with labeled transferrin (red) for 30 min, then immunostained for E-cadherin (green) or (b) co-immunostained for E-cadherin (green) and LAMP1 (red). Arrows indicate colocalization between E-cadherin and transferrin. Nuclei are shown in blue. Insets: areas in *KRT19* KO cells rich in E-cadherin localization. Bar, 20 µm. (c) Parental and *KRT19* KO (KO2) cells treated with dynamin inhibitor (Dynasore) or DMSO control for 2 h were subjected to dispase assay. Data from at least three experimental repeats, each with three replicates, are shown as mean ± SEM. Student’s t-test: ns: not significant; *p < 0.05. (d) Schematic of how K19 influences E-cadherin localization. While E-cadherin is localized to cell surface in the presence of K19 (left panel), it is internalized and accumulated in early/recycling endosomes in the absence of K19 (right panel)

E-cadherin constantly undergoes endocytosis and recycling in the absence of stable cell-cell contacts. The fact that total E-cadherin level is higher while its surface level is lower in *KRT19* KO cells indicates that most E-cadherin is localized in endocytic compartments. To determine the exact location of E-cadherin in *KRT19* KO cells, we performed co-immunostaining of E-cadherin and endocytic markers. Cells were stimulated with labeled transferrin to mark early/recycling endosomes (Figure 4a) while LAMP1 was used as a marker of late endosomes/lysosomes (Figure 4b). Results show that while a subset of E-cadherin colocalized with labeled transferrin in *KRT19* KO cells, very little, if any, E-cadherin colocalized with LAMP1, suggesting that E-cadherin in *KRT19* KO cells localized to early and/or recycling endosomes. Indeed, when *KRT19* KO cells were treated with a dynamin inhibitor dynasore to inhibit E-cadherin internalization, *KRT19* KO cells showed marked improvement in cell-cell adhesion (7.0 vs 2.44 fragments, Figure 4c), suggesting that K19 maintains functional E-cadherin from being internalized (Figure 4d).

### K19 re-expression rescues defects in in KRT19 KO cells

**Figure 5.**
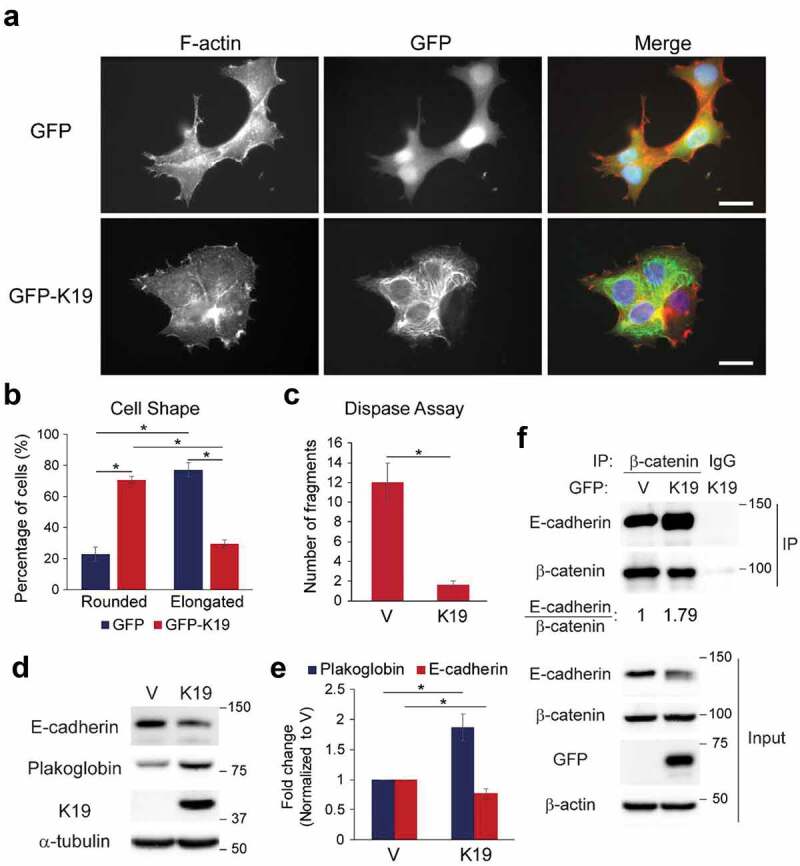
**K19 re-expression rescues defects associated with *KRT19* KO cells**. (a) *KRT19* KO (KO2) cells stably expressing GFP or GFP-K19 were co-immunostained for GFP (green) and F-actin (red). Nuclei are shown in blue. Bar, 20 µm. (b) Percentage of cells from (**A**) with rounded or elongated cell shape. Data from five experimental repeats are shown as mean ± SEM. N = 74 for GFP-expressing cells and N = 115 for GFP-K19-expressing cells. Student’s t-test: *p < 0.005. Chi-square test: p < 0.0001. (c) Number of monolayer fragments formed by *KRT19* KO (KO2) cells stably expressing vector control (v) or K19 from the dispase assay. Data from at least three experimental repeats, each with three replicates, are shown as mean ± SEM. Student’s t-test: *p < 0.05. (d) Whole cell lysates of *KRT19* KO (KO2) cells stably expressing vector control (V) or K19 were prepared and immunoblotting was performed with indicated antibodies. Molecular weights in kDa. (e) Signal intensities of plakoglobin and E-cadherin from (**D**) were quantified and normalized to those of the GAPDH loading control. Data from at least three experimental repeats normalized to that of the vector control are shown as mean ± SEM. Student’s t-test: *p < 0.05. (f) Co-IP of β-catenin with E-cadherin in *KRT19* KO (KO2) cells stably expressing GFP or GFP-K19. IP with anti-β-catenin antibody or IgG control was perform. Immunoprecipitates and inputs were subjected to SDS-PAGE and immunoblotting was performed with antibodies against the indicated proteins. Molecular weights in kDa. Signal intensities of E-cadherin in IP from were quantified and normalized to those of β-catenin in IP and GFP control

To confirm that altered morphology (Figure 1) and cell-cell adhesion (Figure 2) in *KRT19* KO cells were due specifically to the absence of K19, K19 was re-expressed in *KRT19* KO cells. Introducing GFP-tagged K19 in *KRT19* KO cells reverted vast majority of cells into rounded shape (Figure 5a). Quantitation of cell shape, as is done in Figure 1d, confirmed significant increase in cells with rounded shape (70.5 vs 22.9%) upon expression of GFP-K19 in *KRT19* KO cells compared to the GFP control (Figure 5b). Furthermore, *KRT19* KO cells stably expressing K19 were more resistant to dispase-induce fragmentation compared to the vector control (Figure 5c), confirming the role of K19 in maintaining cell-cell adhesion. Re-expression of K19 also raised levels of plakoglobin while lowering levels of E-cadherin (Figure 5d), consistent with altered levels of plakoglobin and E-cadherin observed in *KRT19* KO cells (Figure 3a–Figure 3b). Finally, expression of GFP-K19 in *KRT19* KO cells increased E-cadherin co-immunoprecipitating with β-catenin, suggesting that re-expression of K19 increased formation of the adherens junction complex (Figure 5f). Altogether, these data confirm that K19 is required for proper cell shape and cell-cell adhesion while maintaining plakoglobin level and E-cadherin-β-catenin complex.

### K19 inhibits cell migration and invasion but is required for the anchorage-independent growth

**Figure 6.**
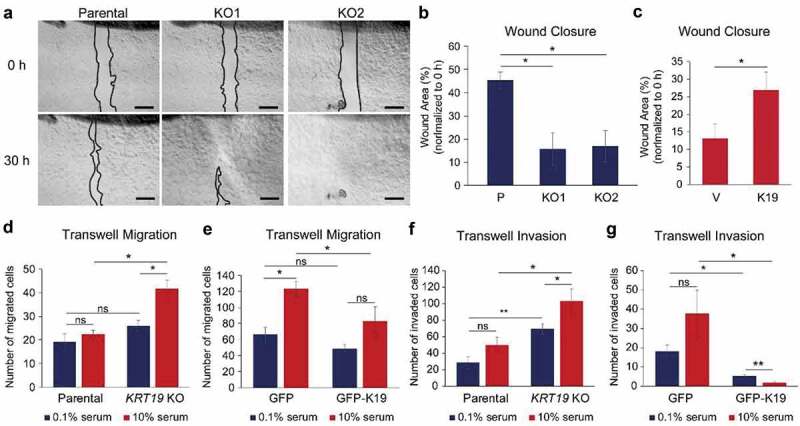
**K19 inhibits cell migration and invasion**. (a) Wound closure of parental (p) control and *KRT19* KO cells. Phase-contrast images of wound area at 0 and 30 h after scratch. Bar, 0.5 mm. (b) Wound areas from (**A**) were quantitated using the ImageJ software and normalized to that at 0 h. (c) Wound closure of *KRT19* KO (KO2) cells stably expressing vector control (v) or K19. Wound areas were quantitated using the ImageJ software and normalized to that at 0 h. Transwell migration of (d) P and *KRT19* KO (KO2) cells or (e) *KRT19* KO (KO2) cells stably expressing GFP or GFP-K19 in the presence of either 0.1 or 10% serum as chemoattractant. Transwell invasion of (f) parental and *KRT19* KO (KO2) cells or (g) *KRT19* KO (KO2) cells stably expressing GFP or GFP-K19 in the presence of either 0.1 or 10% serum as chemoattractant. Migrated/invaded cells were identified either by staining nuclei with propidium iodide or using GFP signals under a fluorescent microscope. For (d-g), Number of migrated or invaded cells per high-power field were quantified the ImageJ software. For (b-g), data from three experimental repeats, each with three replicates, are shown as mean ± SEM. Student’s t-test: ns: not significant; * p < 0.05; **p < 1 × 0.005

**Figure 7.**
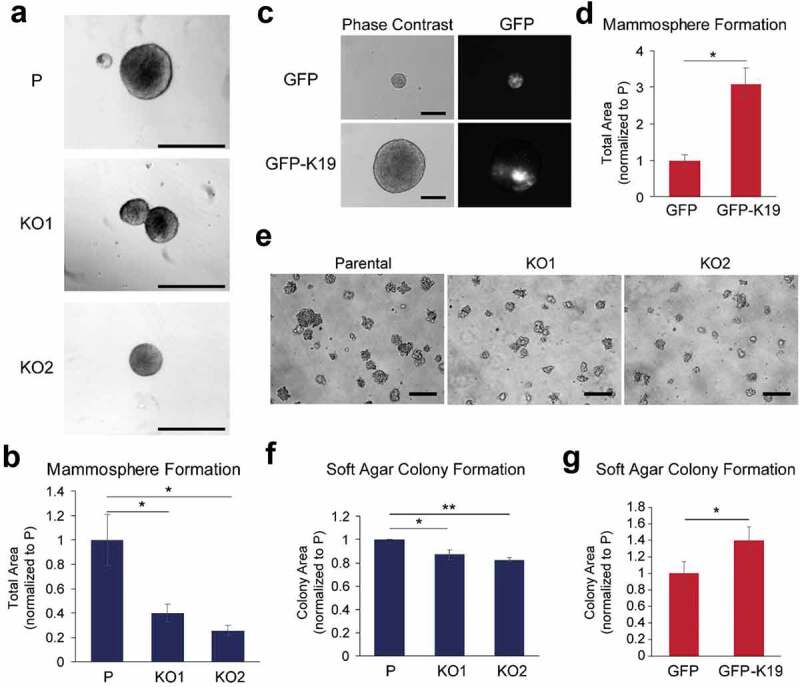
**K19 is required for the anchorage-independent growth of MCF7 cells**. (a) Mammosphere formation of parental (p) control and *KRT19* KO cells. Phase-contrast images of mammospheres grown in ultra-low attachment plates for 7 days. Bar, 100 µm. (b) Mammosphere areas from (**A**) were quantitated using the ImageJ software. (c) Phase contrast and immunofluorescence images of mammospheres formed by *KRT19* KO (KO2) cells stably expressing GFP or GFP-K19 in ultra-low attachment plates. Bar, 100 µm. (d) Mammosphere areas from (**C**) were quantitated using the ImageJ software. (e) Anchorage-independent growth of P and *KRT19* KO cells. Phase-contrast images of colonies grown in soft agar for 7 days. Bar, 200 µm. (f) Colony areas from using (**E**) were quantitated using the ImageJ software. (g) *KRT19* KO (KO2) cells stably expressing GFP or GFP-K19 in soft agar for 7 days and colony areas were measured using the ImageJ software. For (B), (D), (F) and (G), data from three experimental repeats, each with three replicates, are shown as mean ± SEM. Student’s t-test: * p < 0.05; **p < 0.001

Cell morphology and adhesion of cancer cells are linked to events during metastasis such as cell migration, invasion, survival and growth in low adherence conditions. To determine how K19 affects cell migration, wound closure assays were performed. *KRT19* KO cells migrated faster to close scrape wounds compared to parental cells as although 45.4% of wound area was left unclosed by parental cells, only 15.7–17.0% of wound area remained for *KRT19* KO cells 30 h after wounds were made (Figure 6a–Figure 6b). Re-expression of K19 in *KRT19* KO cells slowed wound closure (Figure 6c), confirming the inhibitory role of K19 in cell migration. Transwell migration assays further verified the inhibitory role of K19 in cell migration, as migration was enhanced in *KRT19* KO cells (41.7 cells per high-powered field) compared to parental cells (22.4 cells) when 10% serum was used as a chemoattractant (Figure 6d). Consistently, transwell migration by *KRT19* KO cells was suppressed upon expression of GFP-K19 (Figure 6e). Similar to cell migration phenotypes, *KRT19* KO cells showed increased invasiveness as assessed by invasion through Matrigel-coated transwells (Figure 6f). This increased invasiveness in the absence of K19 was reverted upon GFP-K19 overexpression (Figure 6g). Interestingly however, when cells were placed on low attachment plates to assess growth in suspension, mammosphere formation was compromised in *KRT19* KO cells (Figure 7a–Figure 7b). Re-expression of K19 using GFP-K19 confirmed that K19 is required for mammosphere formation on low attachment plate (Figure 7c–Figure 7d). Similarly, growing cells in soft agar also showed that K19 is responsible for the formation of colonies in anchorage-independent conditions as colony area was decreased in *KRT19* KO cells (Figure 7e–Figure 7f) and reduced formation of colonies was rescued upon GFP-K19 re-expression in *KRT19* KO cells (Figure 7g). Taken together, these data demonstrate that K19 hinders cell migration but promotes colony formation in suspension.

## Discussion

In this study, we demonstrate the role of K19 in maintaining rounded epithelial cell morphology of the MCF7 breast cancer cell line, which is of estrogen-positive luminal subtype where predominant expression of K19 can be found [35–39]. Interestingly, altered cell shape due to changed K19 expression was also observed in transformed mammary stem/progenitor cells [40] and triple negative breast cancer cell lines BT549 [23] and MDA-MB-231 [41], suggesting that K19 plays a crucial role in maintaining the architecture of breast cancer cells across different differentiation stages and molecular subtypes. Although functions of K19 in normal settings have not been fully resolved, K19 is likely involved in maintaining cell architecture through desmosomes as a member of the keratin family of proteins. Still, there are other keratins present in cells to compensate for the loss of K19, and a loss of all keratins did not by itself alter the shape of tumor cells in lung [42]. Therefore, it is rather intriguing that the absence of K19 can have such profound effects on breast cancer cell morphology.

Along with decreased cell-cell adhesion, MCF7 cells lacking K19 showed more migration and invasion, suggesting that the maintenance of cell-cell adhesion by K19 helps cancer cells retain epithelial morphology and inhibits cancer cells from leaving the primary tumor site. Such function would be consistent with the well-known role of keratins during EMT. Of note, vimentin overexpression also caused MCF7 cells to become elongated and more motile [43]. Therefore, K19 may be an integral member of keratins whose levels relative to vimentin govern the morphology and motility of epithelial cells.

Despite faster migration and invasion, *KRT19* KO cells were less efficient in forming mammospheres on low attachment plates and colonies in soft agar, conditions where cells were subjected to growth without being able to adhere to extracellular matrix. Anchorage-independent cell growth has been shown to correlate with metastatic potential as it mimics conditions that circulating tumor cells (CTCs) encounter inside the vasculature [44]. Our findings suggest that strong cell-cell adhesion made by K19 provides survival and growth advantages to cancer cells, thus increases metastatic potential of CTCs. Indeed, it has been shown that clustering of circulating tumor cells confers high metastatic potential [21,45,46], and cell adhesion molecules K14, plakoglobin and E-cadherin have been shown to be required for metastasis [15,21,22]. Therefore, K19 may also be part of breast cancer cell molecular machinery involved in maintaining CTC clusters during metastasis. As our data from migration, mammosphere and soft agar colony formation assays show, K19 seems to either promote or inhibit metastasis in a stage-specific manner. Future studies using an animal model are needed to determine the exact role of K19 in metastasis *in vivo*.

The fact that K19 was required to form colonies in low attachment conditions may help explain interesting observations that have been made previously in regard to tumor metastasis. While the basal-like subtype of breast cancer is considered to be more invasive and aggressive than the luminal subtype [39,47], luminal-like cells without basal-like traits were fully capable of initiating invasive tumors in immune-deficient mice [48]. In fact, phenotypically pure luminal-like breast cancer cells formed larger and more invasive tumors than basal-like cells [48]. Also, increased levels of *KRT19* mRNA in CTCs during metastasis are associated with worse patient survival [49–52], and K19 has been shown to be even released by breast cancer cells of high metastatic potential [53]. Still, as the lack of K19 expression is also correlated with worse survival of young women with triple-negative breast cancer [54], additional studies are needed to reconcile these seemingly conflicting observations. Differences in the role of K19 in tumor metastasis are likely due to the context-dependent role of K19 as breast cancer is a heterogeneous disease and metastasis itself is a complex process involving multiple factors [2,4,6].

K19 is a stem cell marker in several tissue types, including breast tissue [55–57], but its role in cancer stem cells is unclear. As mammosphere formation in low attachment conditions has been linked to cancer stem cell activities due to resistance to anoikis, the requirement of K19 to form mammospheres suggests that K19 plays an active role in maintaining stem-like properties of breast cancer cells. Consistent with our observation, K19-negative mammary progenitor cells showed delayed tumor onset and displayed lower metastatic potential in xenograft assay than K19-positive progenitor cells [26]. As a subpopulation of cancer cells exhibiting stem-like properties are considered to be critical for metastasis [58], detailing the role of K19 in cancer stem cells will be important to study in the future.

In the absence of K19, E-cadherin was found in endocytic compartments indicating that most E-cadherin was internalized in the absence of K19. Indeed, the use of dynamin inhibitor to inhibit internalization of cell surface proteins including E-cadherin strengthened cell-cell adhesion of *KRT19* KO cells, suggesting that internalization of adhesion molecule is a culprit for defective cell-cell adhesion. Still, the mechanism of how K19 affects localization of E-cadherin remains unclear. Without keratins, desmosomes are smaller and consistent with this, plakoglobin levels were dependent on K19. Since a reduction of desmosomes precedes the loss of adherens junctions in tumors [11,59,60], a loss of K19 may internalize E-cadherin by first deregulating desmosomes which would then trigger the disassembly of adherens junctions. However, plakoglobin is also a part of adherens junction and K19 interacts with β-catenin [24]. Therefore, K19 may directly stabilize adherens junction components at the cell surface independent of its effect on desmosomes. Future studies will need to elucidate the exact mechanism of how K19 regulates E-cadherin localization.

E-cadherin has long been considered as a tumor suppressor as it is one key component maintaining the epithelial state [19]. However, recent evidence suggest that E-cadherin is required to promote breast cancer metastasis. While loss of E-cadherin increased invasion, it reduced cell proliferation and survival, CTC number, and metastasis in various models of invasive ductal carcinomas [22]. In addition, knockdown of E-cadherin abrogated mammosphere formation of MCF7 cells [61]. The parallel between K19 and E-cadherin in inhibition of cell invasiveness and promotion of cell proliferation and mammosphere formation [22,32], together with regulation of E-cadherin localization by K19 suggest that K19 may functionally interact with E-cadherin to promote breast cancer metastasis. While our study did not get to determine if increased cell migration and decreased cell proliferation of *KRT19* KO cells are due to decreased cell surface localization of E-cadherin, this study suggests that subcellular localization of E-cadherin may play a critical role in metastasis. Therefore, targeting cell surface E-cadherin may be a promising therapeutic approach for cancer.

## Conclusions

Our study demonstrates that K19 is required to maintain cell morphology and cell-cell adhesion in MCF7 breast cancer cells. Cells lacking K19 show defects in plakoglobin expression, E-cadherin-β-catenin interaction and E-cadherin localization. Importantly, while K19 inhibited cell migration and invasion, it was required to form colonies in low adherent conditions. These data suggest that regulation of cell-cell mechanics of cells by K19 differentially affects processes critical to cancer metastasis.

## Materials and Methods

### Cell lines used

MCF7 *KRT19* KO cells generated using the CRISPR/Cas9 system and MCF7 *KRT19* KO cells stably expressing pLenti CMV/TO hygro empty vector or *KRT19* have been described previously [32]. For MCF7 *KRT19* KO cells stably expressing GFP or GFP-K19, human *KRT19* was cloned out of pMRB101 plasmid (courtesy of Dr. Bishr Omary, Rutgers University) and cloned into pLenti CMV/TO hygro (Addgene, Cambridge, MA) using an In-Fusion HD cloning system (Takara, Mountain View, CA) with oligonucleotides: 5ʹ-TCAGTCGACTGGATCCATGGTGAGCAAGGGCGAGG-3ʹ (BamHI site underlined) and 5ʹ-GAAAGCTGGGTCTAGTCAGAGGACCTTGGAGGCAG-3ʹ (XbaI site underlined), following the manufacturer’s protocol.

### Lentiviral supernatants

Lentiviral supernatants were generated as described previously [32]. Lentiviral supernatants, collected 24 h after transfection, were used to infect subconfluent MCF7 *KRT19* KO cells (KO2) in three sequential 4 h incubation in the presence of 4 µg/ml polybrene (Sigma-Aldrich, St Louis, MO). Transductants were selected in hygromycin (100 µg/ml), beginning 48 h after infection.

### Cell culture and cell lines used

Dulbecco’s Modified Essential Medium (VWR Life Science, Carlsbad, CA) medium was used for MCF7f (ATCC, Manassas VA) growth in 37°C incubator supplied with 5% CO_2_. Medium was supplemented with 10% fetalgro bovine growth serum (RMBIO, Missoula, MT), and 100 units/ml penicillin-100 μg/ml streptomycin (GE Healthcare, Logan UT). MCF7 cells used were authenticated to be 100% match to MCF7 cells (ATCC, HTB-22) by short-tandem repeat profiling service (date performed: 12/18/18). 100 µg/ml hygromycin was added to medium of cells stably expressing pLenti CMV/TO Hygro plasmids.

### Antibodies

The following antibodies: anti-K19 (A-53), anti-K18 (C-04), anti-GAPDH (FL-335), anti-β catenin (15B8), anti-E-cadherin (67A4), anti-β-actin (C4), anti-mouse IgG, and anti-rabbit IgG were from Santa Cruz Biotechnology (Santa Cruz, CA); anti-plakoglobin (D9M1Q) was from Cell Signaling Technology (Danvers, MA); anti-α-tubulin (11,224-1-AP) was from Proteintech (Rosemont, IL); and anti-LAMP1(H4A3) and anti-GFP (12A6) was from the Developmental Studies Hybridoma Bank (Iowa City, IA).

### Cells shape and adhesion assessment

15,000 cells were plated on each well in a 6-well plate. 24 h after plating, at least five random fields of cells in culture were taken using a phase contrast microscope (Olympus optical company. LTD, Japan). To assess cell shape, cells were categorized as either round or elongated. For cell-cell adhesion, cells were categorized into three types (high, medium, or low) based on their attachments to surrounding cells. To quantify number of cells per cluster, cells in contact with adjoining cells were counted a day after passaging. Total of three experiments were analyzed. To measure cell-cell contact lengths, contacts between cells were manually traced using the ImageJ software (National Institute of Health). Minor and major axes as well as circularity were determined by ImageJ after manually tracing outer edges of each cell. Circularity was calculated using the mathematical formula 4π(area)/(perimeter)^2^, and circularity of 1 represents a perfect circle (ImageJ).

### Digital holographic microscope (DHM)

30,000 cells were plated in each 35 mm glass bottom plate. The next day, images were taken using DHM as described previously [34]. The DHM system utilized a 633 nm wavelength HeNe laser to generate reference and object beams and had a lateral resolution of 1.2 µm with a pixel scale of 0.18 µm/pixel. The holograms were captured by a 1.3 MP CMOS camera (Lumenera Corporation, Inc., Ontario, Canada). Detailed information about the set up was published in [34,41,62]. Cell phase-derived pseudoheight maps, calculated directly from phase maps by assuming a cell index of refraction of 1.381, of parental and *KRT19* KO2 cells cultured on glass were collected with a sample size of n = 259 and 173, respectively. Phase images of each individual cell were segmented and 17 phase parameters were extracted (Supplementary Table 1) using an in-house MATLAB code published in [33]. Representative cells were selected by measuring the sum of squared deviations (SSD) of all normalized parameters of each individual cell from the population’s mean, and selecting the cells with the lowest measured SSD values [34].

**Table 1.** Primary antibodies used for immunofluorescence staining

Antibody	Type	Company	Identifier	Stock concentration	Dilution ratio of working solution
anti-K18	anti-mouse	Santa Cruz Biotechnology	C-04	200 µg/ml	1:400
anti-E-cadherin	anti-mouse	Santa Cruz Biotechnology	67A4	200 µg/ml	1:400
anti-GFP	anti-mouse	Developmental Studies Hybridoma Bank	12A6	supernatant	1:500
anti-LAMP1	anti-mouse	Developmental Studies Hybridoma Bank	H4A3	supernatant	1:400

### Dispase assay

Dispase assays were performed as described previously [63]. At 100% confluency, cells in a 6 well plate were washed with 1X PBS then subjected to 2.5 units/ml of dispase (Stemcell Technologies, Kent, WA). The plate was placed on an orbital shaker to induce mechanical stress at room temperature (RT). After 40 min, number of fragments was counted by naked eyes. Images of the plate were taken using ChemiDoc Touch Imager (Bio-Rad, Hercules, CA). Each experiment contained at least three replicates per condition, and every experiment was performed at least three times. For dynasore (Ambeed, Inc, Arlington Heights, IL) treatment, 100 µM of dynasore was added for 2 h in each well before the dispase treatment.

### Preparation of cell lysates, protein gel electrophoresis, and immunoblotting

Cells grown on tissue culture plates were washed with 1X PBS and prepared in cold Triton lysis buffer (1% Triton X-100, 40 mM HEPES (pH 7.5), 120 mM sodium chloride, 1 mM EDTA, 1 mM phenyl methylsulfonyl fluoride, 10 mM sodium pyrophosphate, 1 μg/ml each of cymostatin, leupeptin and pepstatin, 10 μg/ml each of aprotinin and benzamidine, 2 μg/ml antipain, 1 mM sodium orthovanadate, 50 mM sodium fluoride). For immunoblotting, cell lysates were centrifuged at 14,000 rpm for 3 min at 4°C to remove cell debris. Protein concentration was determined using the Bio-Rad Protein Assay (Bio-Rad) with bovine serum albumin (RMBIO) as standard then were prepared in Laemmli SDS-PAGE sample buffer. Aliquots of protein lysate were resolved by SDS-PAGE, transferred to nitrocellulose membranes (0.45 μm) (Bio-Rad, Hercules, CA) and immunoblotted with the indicated antibodies at 1:1000 dilution, followed by horseradish peroxidase-conjugated goat anti-mouse or goat anti-rabbit IgG (Sigma-Aldrich) and Amersham ECL Select Western Blotting Detection Reagent or Pierce ECL Western Blotting Substrate (Thermo Fisher Scientific, Hudson, NH). Signals were detected using ChemiDoc Touch Imager (Bio-Rad) or CL1500 Imaging System (Thermo Fisher Scientific). For western blot signal quantitation, the Image Lab software (Bio-Rad) was used.

### Immunoprecipitation

Immunoprecipitation (IP) was performed as described previously [32]. Cells were lysed using 1% triton lysis buffer. Cell lysates were centrifuged at 14,000 rpm for 3 min at 4°C to remove cell debris, and protein concentrations were measured. Samples were then precleared with prewashed Protein G Sepharose (PGS) beads (GE Healthcare) for 30 min on a shaker at 4°C. Precleared lysates were centrifuged at 14,000 rpm for 3 min at 4°C and beads were discarded. Input sample was prepared and lysates were incubated with indicated antibodies including IgG as a negative control on a shaker for 4 h at 4°C. Samples were then incubated for 45 min with prewashed PGS beads. Afterward, beads were washed 3 times with the lysis buffer and prepared for protein gel electrophoresis and immunoblotting.

### Biotin labeling of cell surface proteins

Biotin labeling of cell surface proteins was performed as described previously [64]. Cell surface proteins were biotin-labeled using sulfo-*N*-hydroxysulfosuccinimide-biotin (Thermo Fisher Scientific) following the manufacturer’s protocol. Cells were washed with ice-cold 20 mM HEPES, pH 7.5 in 1X PBS then treated with 400 μg/ml sulfo-*N*-hydroxysulfosuccinimide-biotin prepared in the washing buffer for 40 min on an orbital shaker at 4°C. A duplicate set of cells was kept without biotin as a negative control. Cells were then lysed with prechilled 1% triton lysis buffer, and cell lysates were centrifuged at 14,000 rpm for 3 min at 4°C to remove cell debris. After measuring protein concentrations, input samples were prepared, and IP was performed with prewashed neutravidin agarose beads (Thermo Fisher Scientific) for 1 h at 4°C. Beads were washed 3 times with the lysis buffer and prepared for protein gel electrophoresis and immunoblotting.

### Immunofluorescence (IF) staining

IF staining of cells was performed as described previously [41]. Cells grown on glass coverslips (VWR) were washed with 1X PBS, fixed in 4% paraformaldehyde in 1X PBS for 35 min, and permeabilized in 0.1% Triton X-100 for 20 min. Samples were blocked in 5% normal goat serum (NGS, RMBIO) in 1X PBS for overnight at 4°C, then stained with primary antibodies diluted in blocking buffer (Table 1) for 16 h at 4°C, followed by Alexa Fluor 488- or 594-conjugated goat anti-mouse secondary antibody (Invitrogen) and 1 μg/ml Dapi (Sigma-Aldrich) with or without 12.5 μg/ml Alexa Fluor 594 Phalloidin (Thermo Fisher Scientific) in 1X PBS for 1 h at RT. After 1X PBS washes, coverslips were mounted on microscope slides with mounting medium containing 1,4-diaza-bicyclo[2.2.2]octane (Electron Microscopy Sciences, Hatfield, PA). Labeled cells were visualized at RT by epifluorescence with an Olympus BX60 Fluorescence Microscope (OPELCO, Dulles, VA) using a UPlanFl 60X NA 1.3, phase 1, oil immersion objective (Olympus). Images were taken with an HQ2 CoolSnap digital camera (Roper Scientific, Germany) and Metamorph Imaging software (Molecular Devices, Sunny Vale, CA). For transferrin labeling, 10 μg/ml of Alexa Fluor 594-conjugated transferrin (Invitrogen) was loaded to cells for 30 min prior to fixation. For E-cadherin and LAMP1 double staining, cells immunostained for E-cadherin with Alexa Fluor 488 secondary antibody were washed in 1X PBS, then blocked with 26 µg/ml of AffiniPure Fab Fragment Goat Anti-Mouse IgG (H + L) (Jackson ImmunoResearch, Inc, West Grove, PA) in 5% NGS in 1X PBS overnight. Cells were washed in 1X PBS before being stained for LAMP1 with Alexa Fluor 594-conjugated goat anti-mouse secondary antibody (Invitrogen).

### Calcium or serum depletion and re-stimulation

Calcium [65] or serum [32] depletion and re-stimulation were performed as described previously. 15,000 cells were plated on each well in a 12 wells plate. Next day, cells were placed in calcium free, low glucose with L-glutamine DMEM (USBiological life science, Salem, MA) or 0.1% serum-containing medium for time indicated in each figure. Cells were then either left unstimulated or stimulated with 5 mM of CaCl_2_ or 10% serum for indicated time in figures. Images of either live cells in culture or cells stained with crystal violet were taken using Olympus CK2 Inverted Trinocular Phase Tissue Culture Microscope (OlympusOptical Co., Japan) equipped with an AM Scope 3.7 digital camera (AmScope, Irvine, CA) [41]. For crystal violet staining of cells, a mixture of 0.1% of crystal violet and 10% ethanol prepared in 1X PBS was added to cells, which were then placed on a shaker at RT for 15 min. Cells were washed with 1X PBS before imaging.

### Wound healing assay

Wound healing assay was performed as described previously [66]. Wounds were made on cells grown to a confluent monolayer on each of a six well plate using a pipet tip. Cells were washed with the medium and images were taken using the light microscope (Olympus) at 0 and 30 h after wounds were made. Percentage of wound closure was calculated by measuring wound area using a free hand tool of the ImageJ software and normalizing area at 30 h to the area of the initial wound at 0 h. Each experiment contained at least three replicates per condition, and every experiment was performed at least three times.

### Transwell migration and invasion assays

Transwell migration [67] and invasion assays [16] were performed as described previously. The Transwell inserts with 8 μm pores (VWR, West Chester, PA) were either left uncoated for migration assay or coated with 40 μl Matrigel (Corning Life Sciences, Corning, NY). Serum-deprived cells in 0.1% serum-containing media were plated in the top chamber for 2 h to allow attachment. 100,000 cells were used for migration assays while 50,000 cells were used for invasion assays. Either 0.1% or 10% serum-containing medium was then added to the bottom chamber. After 48 h, cells were washed, fixed in methanol at – 20°C, and stained with propidium iodide. Cotton swabs were used to remove cells from the top chamber. Fluorescence images of migrated or invaded cells were processed using the ImageJ software to quantify the number of cells. All conditions were performed in triplicates and four high-power fields were imaged per replicate.

### Mammosphere formation assay

Mammosphere formation assay was performed as described previously [61,68]. 100,000 cells were plated on each well of an ultra-low attachment plate (Corning Life Sciences, Corning, NY). After 7 days, images were taken using the light microscope (Olympus). Total area of mammospheres was obtained using the ImageJ software. Each experiment contained at least three replicates per condition, and every experiment was performed at least three times.

### Anchorage-independent growth in soft agar assay

Soft agar colony growth assay was performed as described previously [67]. 20,000 cells were plated per well in a 6-well plate in 1 ml of 0.1% agarose in media on top of a 2-ml bottom layer of 0.5% agarose in media. Plates were incubated at 37°C in incubator for one week. Cells were fed every other day and images were taken using ChemiDoc Touch Imager (Bio-Rad) or CL1500 Imaging System (Thermo Fisher Scientific). Colony area was measured using the ImageJ software. Each experiment contained at least three replicates per condition, and every experiment was performed at least three times.

### MCF7 RNA-sequencing and bioinformatic analyses

RNA-sequencing of parental and *KRT19* KO MCF7 cells was performed previously [32]. List of genes was sorted based on q-value (less than 0.05), and fold change (greater than 1.5). 387 genes upregulated in *KRT19* KO cells were uploaded in functional annotation tool (DAVID software) and keywords in functional categories were selected. Top thirteen pathways were sorted based on gene number.

### Graphs and statistics

Data in bar graphs represent the mean ± standard error of means. For comparisons between two conditions, Student’s t-test was performed to test the statistical significance. When comparing values given in percentages, chi-square test was performed. For experiments involving DHM, all statistical analyses were performed using Systat 13.1 (Systat Software Inc., Chicago, IL). For comparisons between two conditions, the nonparametric Mann-Whitney U-test was performed, with significance set at *p* < 0.05. Before using the test, assumptions of data independence, normality, and homoscedasticity were checked using visual assessment of phase maps (to ensure data were derived from unique cells), the Shapiro-Wilk test, and Levene’s test, respectively. Since most datasets were not normally distributed, the nonparametric test was used. Resulting *p*-values are indicated for select comparisons in Figure 1 and all comparisons in Supplementary Data Table S1. The mean and 95% confidence intervals were placed in Table S1 to facilitate comparisons between parental and *KRT19* KO groups. Representative cell phase maps from digital holographic microscopy were selected based on the minimum sum of squared deviations (SSD) of seventeen normalized phase parameters, as previously described [34]. Briefly, phase parameters from all cell phase maps of either parental or *KRT19* KO cells were standardized using the zscore function in MATLAB vR2015a (The Mathworks, Natick, MA), and SSD calculated for each phase parameter, for each group. A perfectly average cell phase map would have SSD = 0; larger values of SSD imply a cell phase map further from average. All characterizations of cell-cell adhesion from phase contrast and immunofluorescence images were independently evaluated and analyzed by at least two researchers.

## Supplementary Material

Supplemental Material
