# Supplementary material for: Keratin 19 maintains E-cadherin localization at the cell surface and stabilizes cell-cell adhesion of MCF7 cells
Source: Cell Adh Migr. 2021 Jan 4;15(1):1–17. doi: 10.1080/19336918.2020.1868694 (PMC7801129; doi:10.1080/19336918.2020.1868694)
Supplement: Supplemental Material [file KCAM_A_1868694_SM2990.docx]

**Keratin 19 maintains E-cadherin localization at the cell surface and stabilizes cell-cell adhesion**

Sarah Alsharif, Pooja Sharma, Karina Bursch, Rachel Milliken, Van Lam, Arwa Fallatah, Thuc Phan, Meagan Collins, Priya Dohlman, Sarah Tiufekchiev, Georges Nehmetallah, Christopher B. Raub, and Byung Min Chung

**Supplementary Data**

| **Parameter (units)** | **Full Name** | **Parental** | ***KRT19* KO** | ***p*-value** |
| --- | --- | --- | --- | --- |
| PhaseMean (nm)** | Phase height | 3950 (3818,4081) | 3638 (3517,3758) | 0.002 |
| PhaseSD (nm) | Phase standard deviation | 1965 (1892,2038) | 2033 (1953,2113) | 0.12 |
| Ku | Kurtosis | 2.24 (2.16,2.31) | 2.27 (2.13,2.41) | 0.49 |
| sk† | Skew | -0.09 (-0.13,-0.04) | 0.10 (0.05,0.16) | <0.001 |
| Area (μm^2^)† | Segmented cell area | 460 (442,478) | 563 (532,594) | <0.001 |
| Perimeter (μm)† | Segmented cell perimeter | 95 (92,98) | 128 (122,133) | <0.001 |
| Eccentricity† | Cell fit ellipse eccentricity | 0.73 (0.71,0.74) | 0.87 (0.85,0.88) | <0.001 |
| Circularity† | Segmented cell circularity | 0.66 (0.64,0.67) | 0.46 (0.44,0.49) | <0.001 |
| Contrast* | Second-order texture parameters from the segmented cell gray-level co-occurrence matrix | 305 (291,319) | 330 (313,346) | 0.02 |
| Correlation* |  | 0.943 (0.940,0.946) | 0.939 (0.936,0.942) | 0.01 |
| Energy |  | 1.5E-4 (1.4E-4,1.6E-4) | 1.5E-4 (1.4E-4,1.6E-4) | 0.96 |
| Homogeneity |  | 0.184 (0.180,0.188) | 0.182 (0.177,0.186) | 0.68 |
| CentralPhaseMx (nm)† | Maximum phase height, central region | 9108 (8764,9451) | 9530 (9115,9944) | <0.001 |
| CentralPhaseMean (nm) | Mean phase height, central region | 5727 (5531,5924) | 5455 (5264,5646) | 0.08 |
| CentralArea (μm^2^)† | Area, central region | 185 (178,192) | 225 (215,236) | <0.001 |
| Central_ku | Kurtosis, central region | 0.61 (0.59,0.64) | 0.66 (0.62,0.70) | 0.15 |
| Central_sk* | Skew, central region | 1.64 (1.59,1.68) | 1.80 (1.70,1.90) | 0.01 |

**Table S1. Image parameters from segmented phase maps acquired by DHM.** Seventeen geometrical and phase parameters were calculated from segmented phase maps of n = 259 parental and n = 173 *KRT19* KO cells. Image processing details and equations for each parameter are listed in Lam et al., *Cytometry A.* 2019. Mean parameter values for both groups of cells are followed by lower and upper bounds of 95% confidence intervals, in parentheses. P-values from the Mann-Whitney U-test are listed, and parameters with significant differences between groups are marked with * for p < 0.05; ** for p < 0.01; † for p<0.001.

**
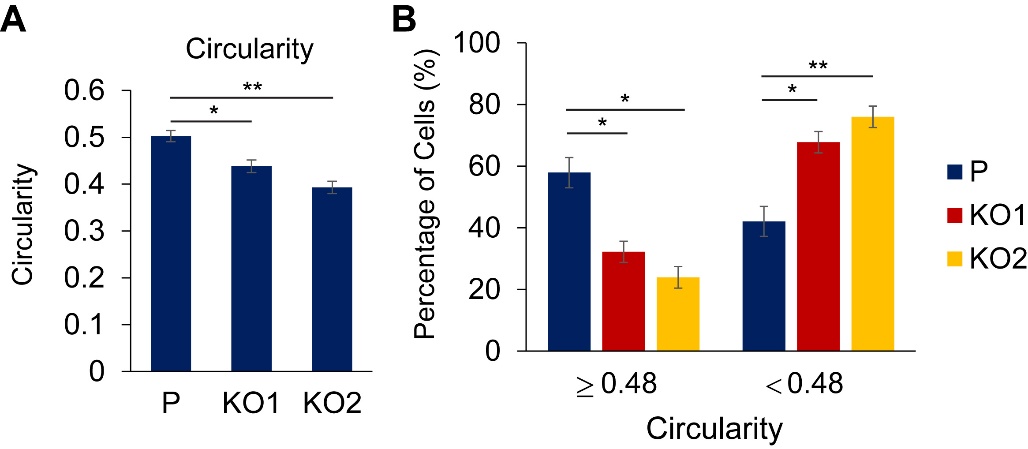
**

**Figure S1. A loss of K19 results in decreased circularity of cells.** (**A**) Circularities of parental (P) and *KRT19* KO cells (KO1 and KO2). Data from four experimental replicates are shown as mean ± SEM. Student’s t-test: *p < 0.05; **p < 0.001. (**B**) Percentages of P and *KRT19* KO cells with circularity greater than or equal to 0.48 or less than 0.48. Each cell was traced Data from four experimental repeats are shown as mean ± SEM. Student’s t-test: *p < 0.05; **p < 0.005. Chi-square test: p < 0.001.

**
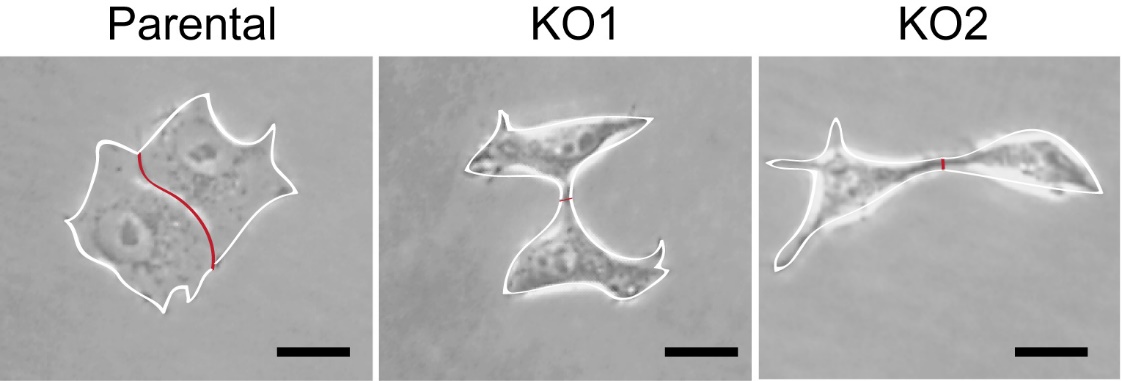
**

**Figure S2. K19 is required for cell-cell contact.** Phase contrast images of parental and *KRT19* KO (KO1 and KO2) cells outlined for cell-cell contacts (in red) and rest of cell perimeters (white). Ratios of cell-cell contact length to cell perimeter are shown in Figure 2E. Bar, 20 µm.

**
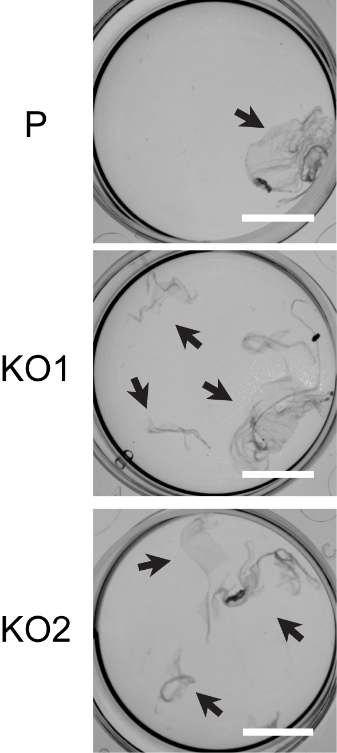
**

**Figure S3. *KRT19* KO cells show weakened cell-cell adhesion.** Phase contrast images of dispase treated parental (P) and *KRT19* KO (KO1 and KO2) fragments of cell sheets (arrows) used for Fig 2E. Bar, 10 mm.

**
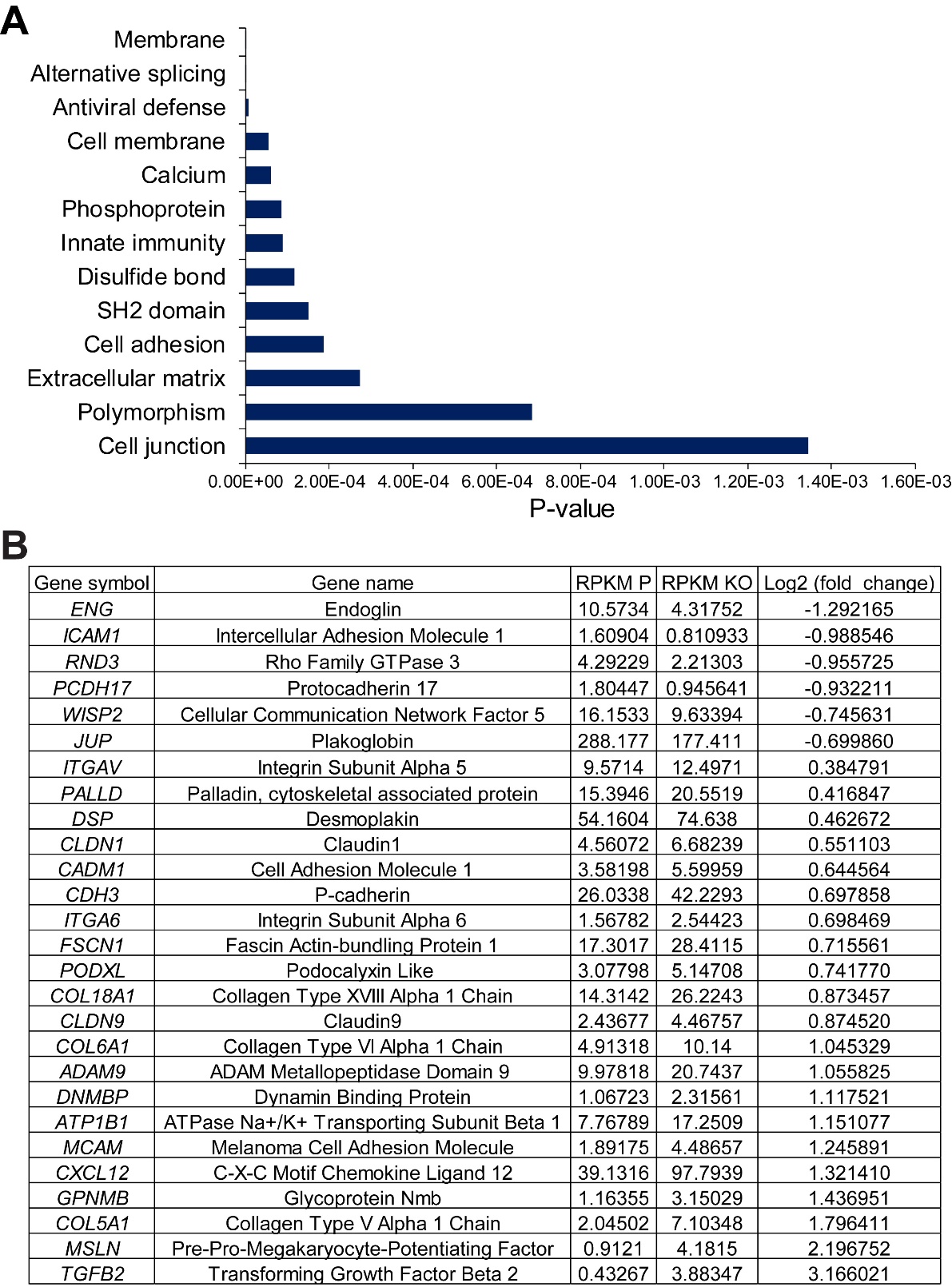
**

**Figure S4. Increased expression of cell adhesion molecules in *KRT19* KO cells.** (**A**) A list of top keywords in functional categories associated with genes upregulated in *KRT19* KO from the RNA sequencing data (Sharma et al., *Sci Reports*. 2019) using the DAVID functional annotation software. (**B**) List of major cell adhesion molecules that are differentially regulated in *KRT19* KO cells.

**
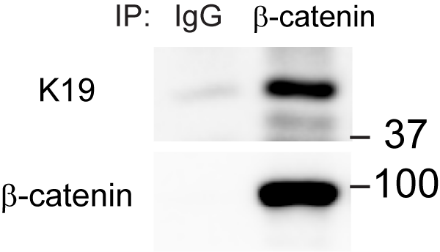
**

**Figure S5. K19 interacts with β-catenin in MCF7 cells.** IP with anti-β-catenin antibody or IgG control was performed. IP samples were subjected to SDS-PAGE and immunoblotting was performed with antibodies against K19 and β-catenin. Molecular weights in kDa.


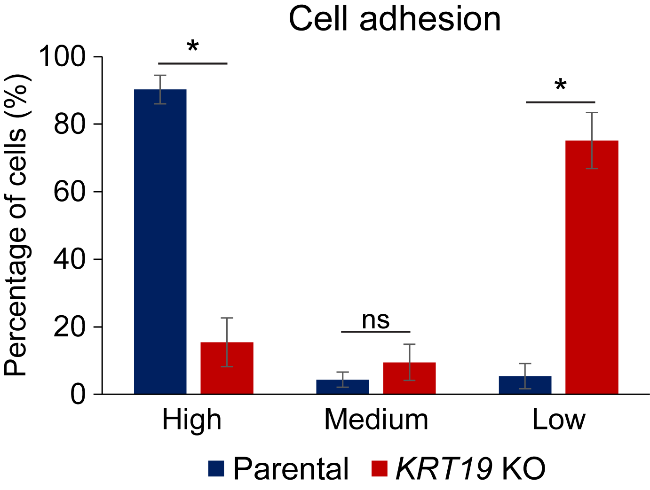


**Figure S6. K19 is required for cells to engage in a high degree of cell adhesion.** Images from Figure 3C were quantified as was done in Figure 2B. Data from at least five experimental repeats are shown as mean ± SEM. N=149 for Parental cells and N=54 for *KRT19* KO cells Student’s t-test: ns: not significant; *p < 1 × 0.0001. Chi-square test: p < 0.0001.

**
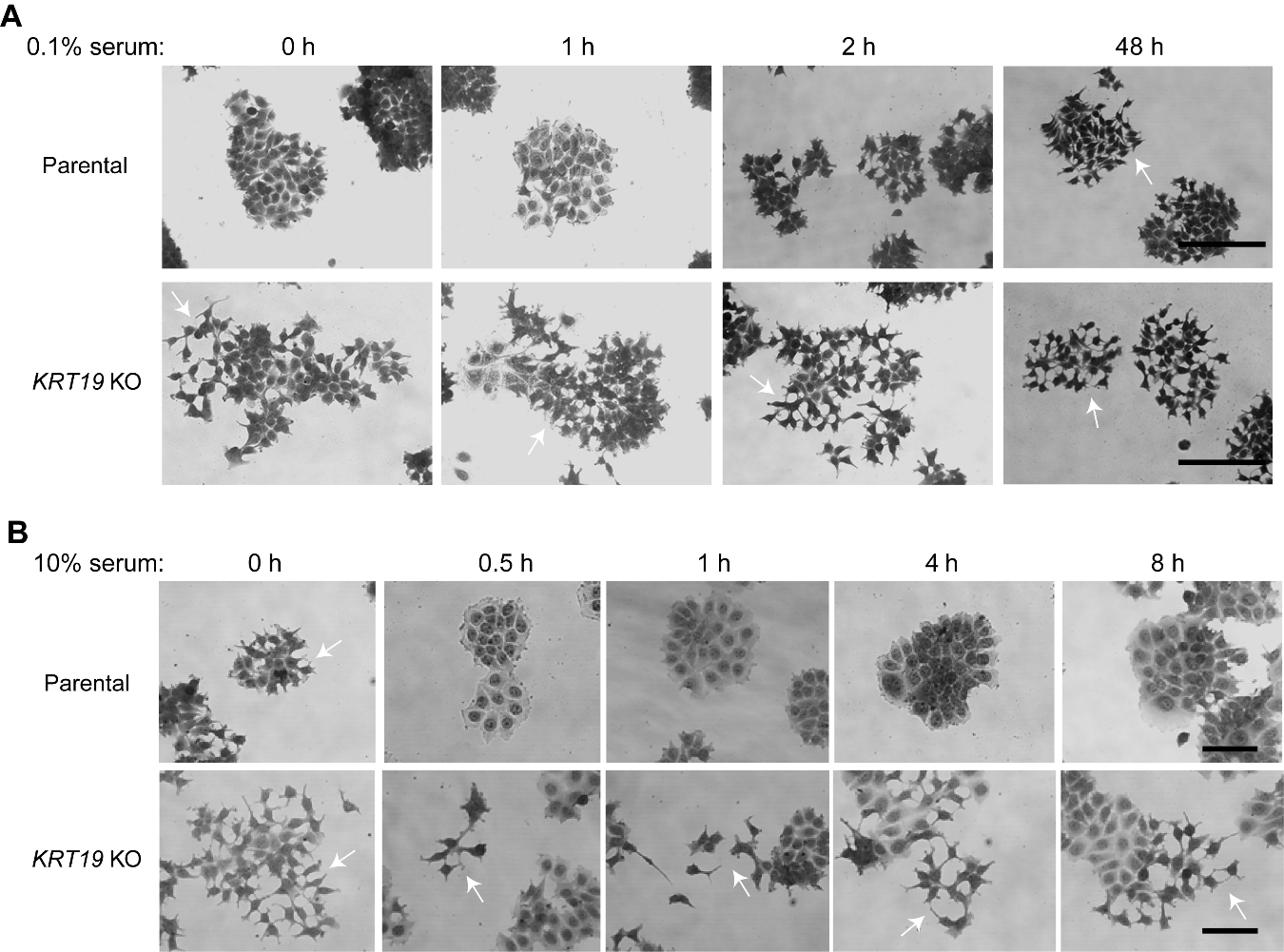
**

**Figure S7. Decreased responsiveness of *KRT19* KO cells for cell-cell adhesion upon serum stimulation.** (**A**) Phase contrast images of parental and *KRT19* KO (KO2) cells following serum starvation for indicated time points. Bar, 200 µm. Whereas parental cells show detachments between cells only after 48 h from serum starvation, shape of *KRT19* KO cells remain detached throughout serum starvation. Bar, 200 µm. (**B**) Phase contrast images of parental and *KRT19* KO (KO2) cells serum-starved for 24 h, then stimulated with 10% serum for indicated time points. Parental cells rapidly re-attach from 0.5 h time point but *KRT19* KO cells remain detached throughout serum starvation. Bar, 100 µm. Bright field images after crystal violet staining were taken, and representative images are shown. Arrows indicate low cell-cell adhesions.
